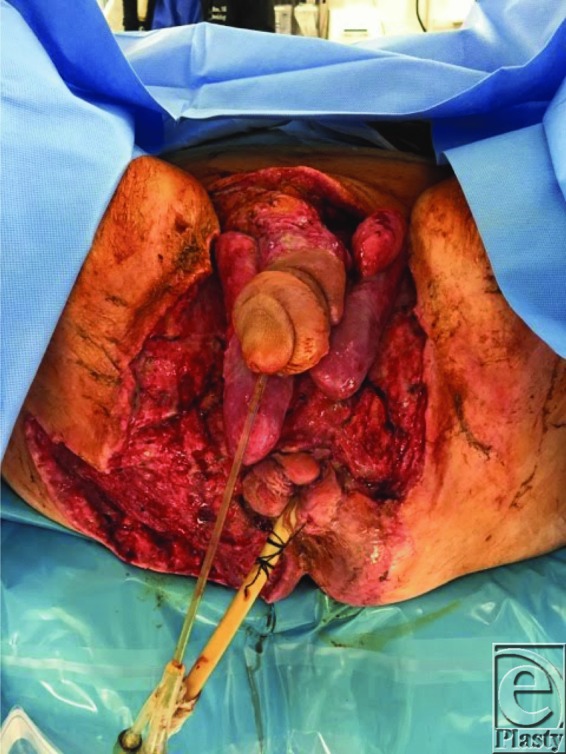# A Case of Fournier's Gangrene

**Published:** 2017-09-18

**Authors:** Anthony Maurice Kordahi, Ahmed S. Suliman

**Affiliations:** Division of Plastic Surgery, University of California San Diego

**Keywords:** Fournier's, gangrene, perineum, systematic review, severity index

## DESCRIPTION

A 57-year-old man presented with a necrotizing soft-tissue infection of the perineum and scrotum in the setting of uncontrolled diabetes mellitus (DM). He was taken initially by the Urology and General Surgery Services for debridement. The case was then turned over to Plastic Surgery for additional debridement and staged reconstruction.

## QUESTIONS

1. What is the epidemiology of Fournier's gangrene?
2. How do patients with Fournier's gangrene commonly present?
3. Are there any predictive scores for the severity of Fournier's gangrene?
4. How are common defects resulting from Fournier's gangrene reconstructed?

## DISCUSSION

Fournier's gangrene is a rare urologic emergency, representing less than 0.02% of hospital admissions in the United States, characterized by progressive necrotizing infection of the external genitalia and/or perineum. Management depends on early recognition, broad-spectrum antibiotics, resuscitation, and aggressive debridement. In a large retrospective review, Sorensen and Krieger^1^ were able to identify 1641 males with Fournier's gangrene. They had an overall fatality rate of 7.5%, mean age of 50.9 years, and more likely to have DM and obesity (odds ratio [OR] = 3.3; 95% confidence interval [CI], 2.9-3.7; OR = 3.7; 95% CI, 2.9-3.7, respectively) than other males. Only 39 women were identified, with similar demographics, but they presented more acutely ill with nonsignificantly longer hospital stays, requirements for mechanical ventilation, and fatality at 12.8%.^1^ Similar review studies have shown higher rates of mortality up to 40%.^2^ Multiple other studies demonstrated congruously elevated mortality rates for women; however, none of these met statistical significance.^3^

In a single-center retrospective review of 43 patients, Oguz et al^4^ described the most common complaint at presentation being perianal/scrotal pain and swelling (72.9%), followed by tachycardia (58.13%), purulent discharge from the perineum (55.81%), crepitus (51.16%), and fever (41.86%). The most common clinical presentation was necrosis in the perineal and genital regions. The most common bacterial organisms found included *Escherichia coli* (48.8%), *Pseudomonas* spp (20.9%), *Enterococcus* spp (18.6%), *Staphylococcus* spp (13.9%), *Streptococcus* spp (11.6%), *Proteus* spp (11.6%), *Acinetobacter* spp (9.3%), *Bacteroides* spp (9.3%), and *Klebsiella pneumoniae* (4.6%).^5^

In 1995, Laor et al developed a Fournier's gangrene severity index (FGSI) to assess severity of disease. This is the most widely accepted index score for stratifying severity of Fournier's gangrene.^7^ The 9 parameters are graded on a 0 to 4 scale and include heart rate, temperature, respiratory rate, sodium and potassium levels, creatinine and bicarbonate levels, hematocrit, and leukocyte count. Patients with FGSI score of more than 9 had a 75% probability of mortality.^6^ Lin et al^5^ generated a simplified FGSI score from a sample of 85 patients that included only creatinine, hematocrit, and potassium. They obtained a sensitivity and specificity of 87% and 77%, respectively.

A systematic review of 425 patients performed by Karian et al^6^ discussed the varied methods of wound closure. In order of least to most common, the methods were as follows: 1.4% of defects closed with loose wound approximation, 5.2% with a combination of flaps grafts and tissue adhesives, 5.9% allowed to heal by secondary intention, 8.5% with implantation of the testicle in a medial thigh pocket, 10.4% with delayed primary closure, 16% with scrotal advancement flaps, 22.6% with skin grafts, and 30.1% with flaps. For patients with defects confined to less than 50% of the scrotum, the method of closure was secondary intention, loose approximation/primary closure, and local scrotal advancement flaps.^6^ For larger defects, use of split-thickness skin grafting provided adequate coverage, with minimal adhesion formation that resolved at 6 months with the use of skin emollients and massage.^7^ Flap coverage was used in patients with more extensive defects, the most common being medial thigh fasciocutaneous flaps, pudendal thigh flaps, gracilis flaps, anterolateral flaps, and medial circumflex femoral artery perforator flaps.^6^

In summary, Fournier's gangrene is a rare yet very morbid necrotizing infection of the perineum. It more commonly affects males; yet, affected females tend to present with more severe symptoms and have a statistically nonsignificant higher rate of mortality, potentially due to small sample size. Prompt aggressive debridement, intravenous antibiotics, and resuscitation are the criterion standard initial treatment. Depending on the resulting defect, there are various reconstructive options in the plastic surgeon's armamentarium to provide adequate coverage.

## Figures and Tables

**Figure 1:**
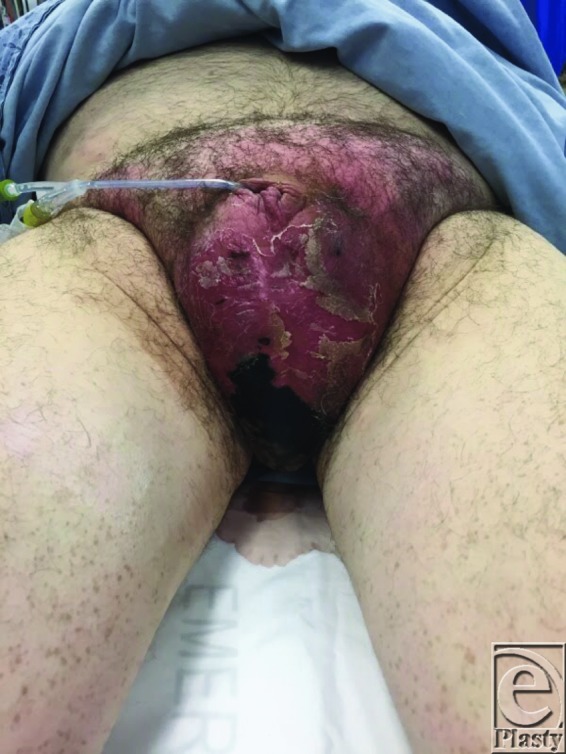


**Figure 2:**
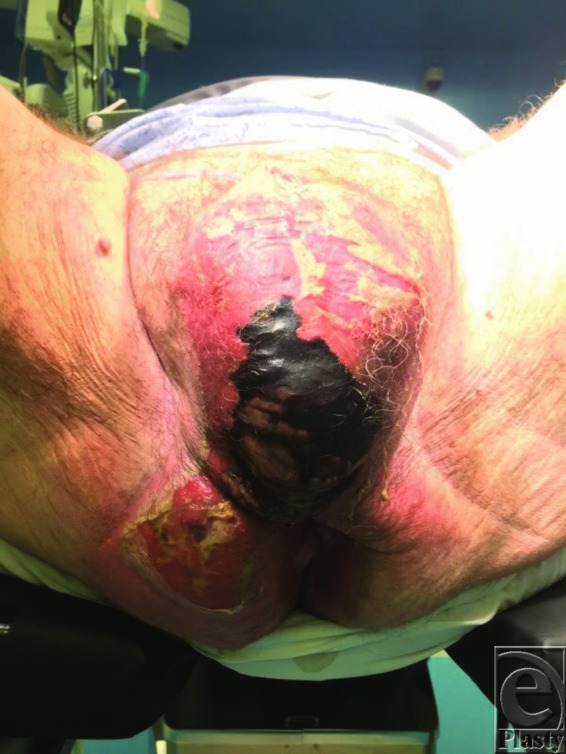


**Figure 3:**